# Comparative Analysis of VOCs from Winter Melon Pomace Fibers before and after Bleaching Treatment with H_2_O_2_

**DOI:** 10.3390/molecules27072336

**Published:** 2022-04-05

**Authors:** Laura Maletti, Veronica D’Eusanio, Caterina Durante, Andrea Marchetti, Luca Pincelli, Lorenzo Tassi

**Affiliations:** 1 Department of Chemical and Geological Sciences, University of Modena and Reggio Emilia, 41125 Modena, Italy; veronica.deusanio@unimore.it (V.D.); caterina.durante@unimore.it (C.D.); andrea.marchetti@unimore.it (A.M.); 217063@studenti.unimore.it (L.P.); lorenzo.tassi@unimore.it (L.T.); 2 Consorzio Interuniversitario Nazionale per la Scienza e Tecnologia dei Materiali (INSTM), 50121 Firenze, Italy; 3 Interdepartmental Research Center BIOGEST-SITEIA, University of Modena and Reggio Emilia, 42124 Reggio Emilia, Italy

**Keywords:** dietary fibers, food waste, HS-SPME-GC-MS, volatile compounds, winter melon, recycle, nutrient recovery, biorefinery

## Abstract

In this study, the trend of Volatile Organic Compounds (VOCs) in dietary fiber samples from the winter melon (*Cucumis Melo* var. *Inodorus*, Yellow Canary type) were investigated. This foodstuff, obtained as a by-product of agri-food production, has gained increasing attention and is characterized by many bioactive components and a high dietary-fiber content. As regards fiber, it is poorly colored, but it may be whitened by applying a bleaching treatment with H_2_O_2_. The result is a fibrous material for specific applications in food manufacturing, for example, as a corrector for some functional and technological properties. This treatment is healthy and safe for consumers and widely applied in industrial food processes. In this study, a method based on headspace solid-phase microextraction (HS-SPME) coupled with gas chromatography–mass spectrometry (GC-MS) was applied for the characterization of the aromatic profile of the dried raw materials. Furthermore, VOC variation was investigated as function of the bleaching treatment with H_2_O_2_. The bleached samples were also analyzed after a long storage period (24 months), to assess their stability over time. As a result, the VOC fraction of the fresh raw fiber showed nine classes of analytes; these were restricted to seven for the bleached fiber at t_0_ time, and further reduced to four classes at the age of 24 months. Alcohols were the main group detected in the fresh raw sample (33.8 % of the total chromatogram area), with 2,3-butanediol isomers as the main compounds. These analytes decreased with time. An opposite trend was observed for the acids (9.7% at t_0_), which increased with time and became the most important class in the 24-month aged and bleached sample (57.3%).

## 1. Introduction

The global market for dietary fibers (DF) represents a fast-growing and rapidly expanding commercial sector, always on the lookout for new possibilities and technological applications. This is quite evident considering their use as formulation ingredients in baked goods, ice cream, and pre-packaged foods, while they also play functional roles as stabilizers, gelling agents, adjuvants, etc. In fact, in several well-documented reports, including those of Food and Agriculture Organization of the United Nations (FAO), increased consumption of dietary fibers is strongly recommended in the human diet [1,2,3]. These suggestions are mainly aimed at populations that have adopted a Western lifestyle, with a diet typically high in protein, lipid, and carbohydrate consumption. Such a diet is not particularly healthy due to the low daily intake of fibers [4]. Moreover, in a context where forced food production rates are becoming less and less sustainable globally [5], and where the amount of unused food is growing exponentially [6], the ability to obtain useful products from waste materials is also of utmost importance from a circular economy perspective. Furthermore, world hunger is a condition that afflicts about 700 million people, and a series of indicators from multidimensional measurement of the problem suggests that about 2 billion people face moderate or severe levels of food insecurity [7]. In order to maximize the spillover effects due to agronomic overproduction, it is necessary to raise awareness among producers, consumers, industrial processors, and stakeholders [5]. In this way, at the same time, it is possible to mitigate anti-health effects due to both the overconsumption and deprivation of food. In fact, the problem of waste and loss of food can become a source of unexpected resources if compared to the most common current behavioral models.

This framework gives rise to the need to recover as many nutritional components as possible from agro-industrial by-products and from residual product from primary agronomic productions [8]. The latter refers to the massive quantity of fruit and vegetable products that remain on the crop field in a pre-waste condition, i.e., abandoned and not harvested due to overproduction, over-ripening, or for aesthetic reasons that do not comply with quality standards (deformed products, non-commercial sizes, etc.). Among the agri-food products particularly rich in fibers, and widespread across most of the globe, *Cucurbitaceae* have always occupied a prominent position [9]. In order to deepen knowledge of the enhancement of these crops, in this study, particular attention has been paid to the dietary fiber obtainable from melon (*Cucumis Melo* var. *Inodorus*, Yellow Canary type).

In some recent studies, a broad overview of the compositional characteristics of several melon cultivars, to some extent representative of the world production of these fruits, has been provided [10,11]. Till now, the aromatic profile of non-climacteric Inodorus melons has received little attention in the literature, even though these melons have great interest as fresh-cut or processed fruits thanks to their longer shelf-life than climacteric ones [12]. Despite the name *Inodorus*, the aromatic profile of these fruits has been shown to be quite consistent and varied in composition [13]. This aspect could represent the best empirical basis on which to favor and optimize the cultivation and the consumption of this type of melon. The volatile fraction of food is strictly connected to its quality. Indeed, the aromatic profile is one of the most appreciated characteristics by consumers, and Volatile Organic Compounds (VOCs) play a key role in determining the perception and acceptability of products [14]. In addition, being closely related to the chemical composition, the volatile fraction provides information about the constituent components of food [15]. For these reasons, flavor analysis is performed to understand the nature of existing molecular species, to study shelf-life, and to maintain, as much as possible, the quality of foods.

In this study, the VOC fraction of the pomace fiber extracted from Yellow Canary melon type (hereafter called ‘winter melon’) was investigated. In particular, both Melon pomace ‘As it Is’, Vacuum-freeze-dried (M_AI_V), and the Oven-dried Bleached fiber (M_B_O), obtained through the process described below, were taken into account. This choice allowed us to characterize the aroma of the starting material and to monitor VOC variations as a function of the bleaching treatment. In this way, it was possible to evaluate the variation in the fiber quality as well as the aromatic profiles. Furthermore, M_B_O stored for a long time (24 months) was also analyzed, in order to investigate a possible use for these fibers as a functional ingredient within foodstuffs belonging to the above-mentioned categories. Aging of food can be described as the natural reduction of the initial characteristics during storage. It causes composition changes and alterations due to several unwanted but unavoidable processes such as oxidation, photolysis and thermal decomposition. In addition to the action of chemical and physical agents that increases the instability of the fibers, other negative effects may be present. These may include the presence of bacterial strains, capable of metabolizing some analytes, or classes of compounds present at the initial time (t_0_) of fiber preparation.

Bearing this in mind, in this study, the whole dried melon pomace fiber was chemically characterized as well, following the evolution of the VOCs of the bleached fibers over two years. One of the main aims of this framework was to identify some markers associated with the conservation status and aging level of the investigated material. Unfortunately, the study of the evolution of the AI fiber VOCs over two years was not possible since the residual humidity and the sugar content allowed the formation of mold. As a result, the healthiness of the product has been lost.

Nevertheless, H_2_O_2_-bleached fiber is much more interesting as a functional ingredient for potential industrial applications. It is absolutely colorless, with almost neutral organoleptic properties, and retains chromatic stability even after a long aging period. Therefore, the bleaching procedure allows the exploitation of this fiber for any type of application that does not require a specific color or aromatic contribution. In fact, several sources with a high DF content have a limited field of application due to their natural coloring or residual aroma [16,17]. The use of hydrogen peroxide for these purposes is already known in several works [16,17,18], with it being a chlorine- and sulfite-free bleaching agent. Furthermore, it is perfectly bio-compatible, with water being the reduction product.

## 2. Results

The first aim of this research was to develop a straightforward methodology, to provide effective characterization of the VOC aromatic profile of dietary fiber recovered from pre-waste melon fruits. Furthermore, from this study, we aimed to obtain a suitable screening method for sampling VOCs from melon pomace and DF at different aging steps. By extending the study to other crop varieties, it could be possible to produce a robust correlation between the characteristics of the cultivar and the shelf life of the DF itself.

**Figure 1:**
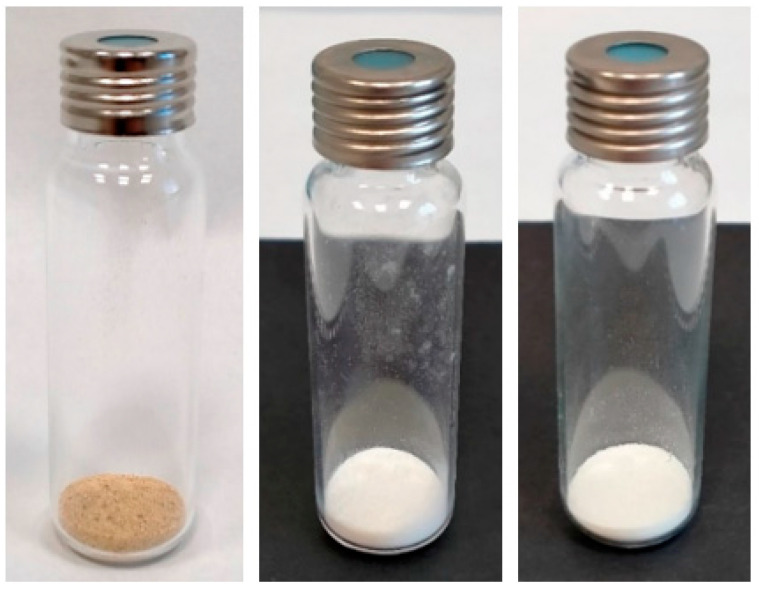
Images of the M_AI_V_t_0_ (**left**), M_B_O_t_0_ (**middle**) and M_B_O_t_24_ (**right**) samples from winter melon pomace fiber.

Figure 1 shows the vacuum-freeze-dried melon pomace “As-it-Is” at t_0_ time (M_AI_V_t_0_), and the bleached fiber immediately after the H_2_O_2_ treatment (M_B_O_t_0_) and after a storage period of 24 months in the closed vial, in the dark, and at room temperature (M_B_O_t_24_).

**Figure 2:**
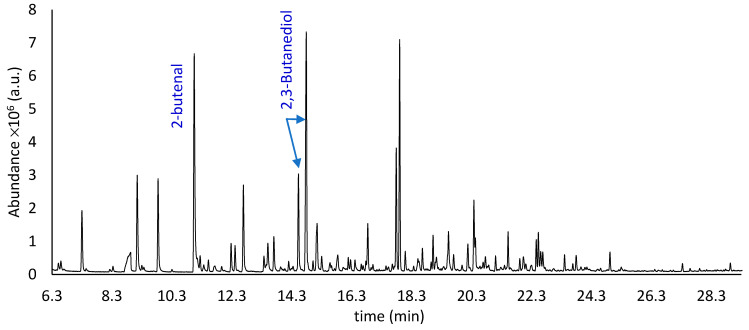
Total ion current chromatogram of VOCs from the M_AI_V_t_0_ fibrous sample, obtained using HS-SPME-GC-MS (a.u. = arbitrary units).

Figure 2 shows the HS-SPME chromatogram obtained from the vacuum-freeze-dried melon pomace “As it Is”, immediately processed with the GC-MS instrumentation (t_0_ time), and Table 1 summarizes the identified compounds.

From Table 1, in the M_AI_V_t_0_ sample, 81 compounds are detected, including 19 aldehydes (ALD); 21 alcohols (ALC); 9 esters, among which 5 were non-acetate esters (NAE) and 4 were acetate ones (ACE); 11 ketones (KET); 8 acids (ACD); 2 alkanes (AHA); 6 sulfur-derived compounds (SDC); and 5 other compounds (OTH).

**Figure 3:**
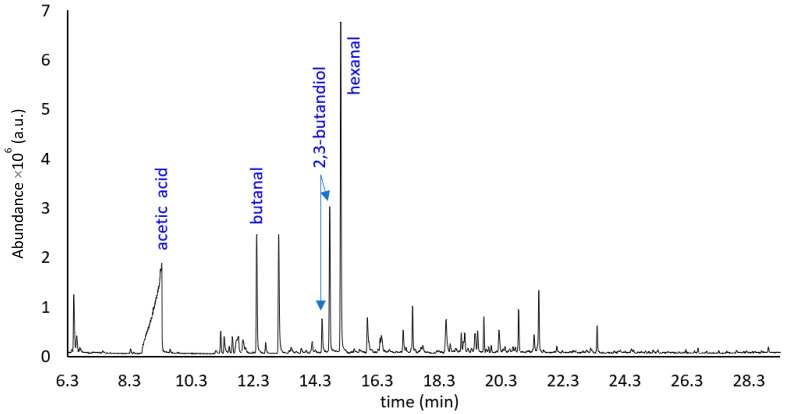
Total ion current chromatogram of the VOCs from M_B_O_t_0_ fibrous sample, obtained using HS-SPME-GC-MS (a.u. = arbitrary units).

Figure 3 shows the HS-SPME chromatogram related to the oven-dried bleached fiber from the winter melon pomace (M_B_O_t_0_). These specimens were immediately processed with the GC-MS instrumentation described in Section 4.

At first sight, the comparison between the chromatograms in Figure 2 and Figure 3 shows a lower number of peaks for the sample that was bleached and oven dried. Evidently the bleaching process and the washing steps remove significant quantities of some volatiles.

Table 2 collects the results of the HS-SPME-GC-MS analysis of the winter melon pomace sample that was bleached with H_2_O_2_, oven dried and analyzed immediately after its preparation.

For the bleached fiber, at t_0_ time (M_B_O_t_0_), 50 VOCs are identified, among which were 16 aldehydes (ALD), 8 alcohols (ALC), 2 non-acetate esters (NAE), 11 ketones (KET), 5 acids (ACD), 3 alkanes (AHA) and 5 other compounds (OTH). Finally, in M_B_O_t_24_, only 30 volatiles are determined and are classified into 4 groups: 9 ACD, 8 ALD, 7 KET and 6 ALC, as reported in Table 3.

**Figure 4:**
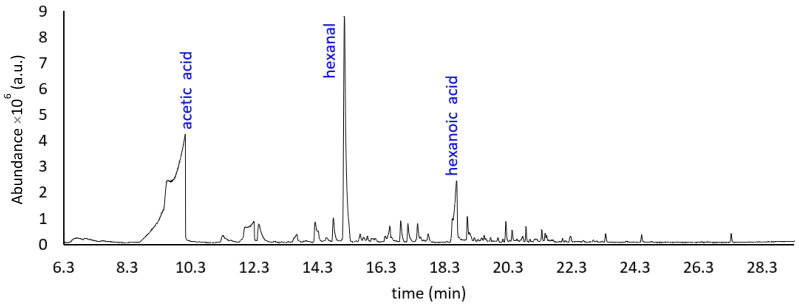
Total ion chromatogram of the VOCs from M_B_O_t_24_ fibrous sample, obtained using HS-SPME-GC-MS analysis (a.u. = arbitrary units).

Figure 4 shows the HS-SPME-GC-MS chromatogram of a sample of bleached melon pomace fiber, oven dried and stored for 24 months in a closed vessel, in the dark, at room temperature. Table 3 reports its descriptive features.

## 3. Discussion

Although winter melon belongs to the *Inodorus* group, it is still possible to note, from Figure 2 and Table 1, a considerable richness and complexity of the aromatic profile of the pomace fiber (M_AI_V_t_0_). This volatile fraction is mainly characterized by very pleasant green, fresh, sweet, fruity and floral notes. This bouquet undergoes significant modification due to the bleaching process (Figure 3, Table 2) applied to the fiber (M_B_O_t_0_). This treatment leads to a substantial increase in the amounts of some analytes, such as acetic and propanoic acid, as well as the appearance or disappearance of some others, such as C9 alcohols and sulfur-derived compounds. Obviously, the natural aging process of the fiber (Figure 4, Table 3) also leads to a change in the composition of the aromatic fraction, which, after 24 months, is considerably depleted of many of its original molecules.

As mentioned throughout the text, we are looking for DFs that are stable over time and as neutral as possible in terms of color, aroma and flavor. Although we are aware that fewer VOCs do not directly correspond to the absence of odors and aromas, our findings represent a very interesting and encouraging result for the purpose of our study. Below are the most significant and representative experimental results of the detected VOCs in the analyzed samples. The different compounds are separately discussed based on their chemical class.

### 3.1. Aldehydes

Aldehydes (ALD) are known to be key compounds that define the flavor and aroma profile of fruits and vegetables. These analytes are usually present in higher concentrations in non-ripe melon cultivars, then are gradually replaced by esters and alcohols as the degree of ripeness progresses [28]. Therefore, most of them (especially C*n* with *n* < 8) are not detected or are only recovered in very small quantities in ripe fruits [32]. Nevertheless, Güler et al. [32], and Shi et al. [10] also reported that aldehydes were the most abundant compounds in some *Inodorus* melon cultivars. The results obtained in the present study are in accordance with literature information since, in this case, aldehydes also represent a very important family of the VOC fraction of the M_AI_V_t_0_ fiber (≈25%), together with alcohols (≈34%). This is the reason that *Inodorus* melons are attributed the lowest odor perception but the highest freshness score, generally being characterized by a “green-like” and herbaceous aroma [32].

Previous studies suggest that saturated and unsaturated C9 aliphatic aldehydes, together with C9 alcohols, play a key role in melon and watermelon aroma [10,29,39]. For melons belonging to the *Inodorus* group, the main analytes playing this role are (*Z*)-6-nonenal, nonanal, (*E*,*Z*)-2,6-nonadienal, (*E*)-2-nonenal, 6-nonen-1-ol and 1-nonanol [10]. All these molecules contribute, to some extent, to imparting waxy, fresh and green notes to the smell of winter melon. Among these aldehydes, we detected only nonanal, both in samples M_AI_V_t_0_ and M_B_O_t_0_, in the amounts of 3.16 × 10^7^ and 30.5 × 10^6^, respectively (TIC peak area); this confirms that this analyte is particularly representative of the DFs and persists even after the strong invasive treatment with H_2_O_2_.

The other aldehydes were not detected, probably due to many factors, including their very low quantity in our samples (below the detection limit), the selected cultivar, origin and cultivation practices, fruit ripening, handling, and post-harvest processing. All these variables can influence the concentration of VOCs in melons [30], altering their aromatic profile. Among the aldehydes, we observed that 2-butenal and 2-methyl-2-butenal were also present in rather high levels in the HS of M_AI_V_t0 (TIC = 19.8 × 10^7^ and 1.28 × 10^7^, respectively). However, only the former persisted after the H_2_O_2_ bleaching process (Table 2), albeit in quantities (2.03 × 10^6^) about 100 times lower than the value of the fresh raw material.

**Figure 5:**
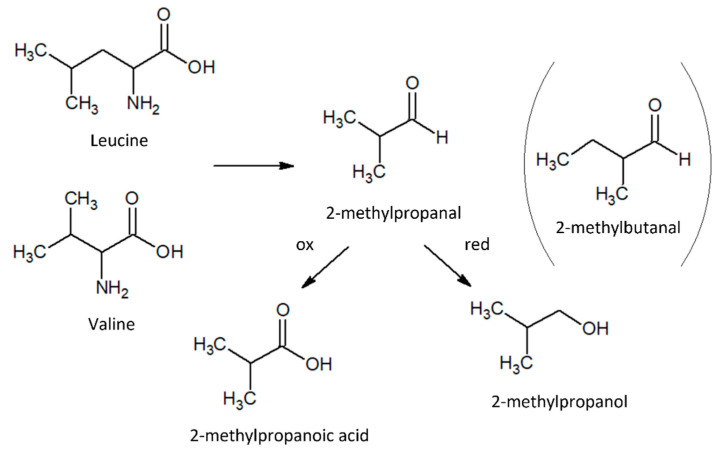
Scheme of 2-methylpropanal biosynthetic pathway and red-ox molecular evolution.

Pentanal and heptanal, on the other hand, showed exactly the opposite behavior, increasing their concentration by about three to four times, respectively, immediately after the treatment of the fiber with H_2_O_2_ (M_B_O_t_0_). During the aging of the DF, these two aldehydes were partially converted to their most oxidized form, namely pentanoic and heptanoic acid, respectively. This may be due to radical oxidation processes initialized by oxygen present in the residual atmosphere inside the vials storing the samples. The branched-chain molecules 2-methylbutanal and 3-methylbutanal, described as having a ‘malty-like’ aroma, were detected in all three of the different samples analyzed. These compounds are suggested to be synthesized from isoleucine and leucine, respectively, even though the pathways are not well recognized yet [30]. However, which fraction of the total quantity of 2-methylbutanal and 3-methylbutanal is not directly related to the aforementioned amino acids cannot be established. It is assumed that 2-methylpropanal is also an intermediate product in the catabolism of two amino acids (leucine and valine); together with 2-methylbutanal, it is considered a key compound in several foods, including melon. Following the literature suggestions, it should, therefore, be converted into parental compounds—the oxidized one, 2-methylpropanoic acid, or the reduced one, 2-methylpropanol [19] (Figure 5).

Six-carbon aldehydes, such as hexanal and (E)-2-hexenal, as well as their corresponding alcohols and straight-chain C9 compounds, such as (*E*,*E*)-2,6-nonadienal and (*E*,*Z*)-2,6-nonadienal, are typically produced by lipoxygenase activity in damaged plant tissues [26,30]. For this reason, these compounds increase in number and concentration after the disruption of cells and tissues. These observations perfectly match our results, as these analytes were absent in the M_AI_V_t_0_ sample. In addition, hexanal was detected in both bleached samples, becoming the aldehyde present in the greatest quantity compared to all the others. In fact, in the sample M_B_O_t_0_ (Table 2), the hexanal had a peak TIC area of 155 × 10^6^, corresponding to a relative area of 43% within the class of aldehydes. In the M_B_O_t_24_ sample, it reached a quantity of 448 × 10^6^ (Table 3), that is, almost 72% of all aldehydes. It conferred grassy, green-like, and fatty notes to the aroma of our bleached fibers. This behavior may have been caused by the synergistic effect of the damage to the plant tissues and the oxidative action of H_2_O_2_ towards the molecules originally present in the matrix. In fact, hexanal is used as an index for the degree of oxidation of fatty acids in food chemistry [32].

Among the identified aromatic aldehydes, benzaldehyde, benzeneacetaldehyde and α-ethylidene-benzeneacetaldehyde, were detected, albeit in small concentrations. They covered only about 2% of the entire volatile fraction of the vacuum-freeze-dried melon pulp (Table 1). In particular, benzeneacetaldehyde was the only one initially identified both in the M_AI_V_t_0_ fiber and after the bleaching treatment (M_B_O_t_0_), but none of the three molecules were preserved during the aging period. Unlike what is reported by Güler et al. [32], in our case, the presence of acetaldehyde was not detected, probably for the same reasons previously suggested for the C9 aldehydes. Furthermore, it is important to consider that most of the studies found in the literature refer to “fresh” fruit pulp, or juice, and not to dehydrated material. This aspect certainly contributes to the differences between this study and previous ones. On the other hand, to the best of our knowledge, this is the first time that *α*-ethylidene-benzeneacetaldehyde (2-phenyl-2-butenal) has been mentioned among the melon VOCs. For this reason, it is quite difficult to highlight its contribution in terms of perceived aroma. Finally, in all three samples, furfural was identified. It is a natural dehydration product of five-carbon sugars, such as xylose and arabinose, which are typically found in the hemicellulose fraction of fruit and vegetables [40]. In particular, its amount, expressed as the TIC peak area, increased by about four times from the M_AI_V_t_0_ sample (0.634 × 10^7^, Table 1) to the M_B_O_t_0_ (26.6 × 10^6^, Table 2) one. After the long aging period, the DF presented a furfural amount of 9.24 × 10^6^ (Table 3).

### 3.2. Alcohols

Alcohols were the most abundant analytes in the HS of the M_AI_V_t_0_ sample, both in terms of the number of analytes (21) and quantity, since they covered about the 34% of the total VOC fraction. In particular, among them, 2,3-butanediol was the most abundant (≈49%, considering the sum of the two stereoisomers), together with benzyl alcohol (≈8.7%), 3-pentanol (≈9.4%) and 1-octen-3-ol (≈4.3%) in the above-mentioned sample (Table 1). They conferred to the aromatic profile a variety of creamy, buttery, fruity, sweet, and earthy notes. Benzyl alcohol is a very important volatile compound for the melon aroma, with its sweet and fruity contribution. This molecule has already been identified in many different cultivars, even though it has not been not present in high quantities [10]. 2,3-butanediol was identified as the major volatile compound in banana fruit [41]. It was detected in two isomers in our study, (*R*,*R*)- and (*R*,*S*)-2,3-butanediol. It was found in all three samples analyzed, but its quantity was drastically reduced after the bleaching treatment with H_2_O_2_, passing from 2.74 × 10^8^ in M_AI_V_t_0_ (Table 1), to 9.08 × 10^7^ (≈−67%) in M_B_O_t_0_ (Table 2), and finally, to 6.24 × 10^7^ after 24 months of storage (≈−77% compared to the fresh raw DF).

Its presence is related to butanedione and 3-hydroxy-2-butanone (acetoin), as will be discussed in the subsection dedicated to the category of ketones. As already mentioned for aldehydes, aliphatic C9 alcohols have also been previously described as the main typical odorants in melon and watermelon fruits. For the latter, 3,6-nonadienol; 3-nonen-1-ol; 6-nonen-1-ol; and 1-nonanol are the prevailing compounds of this class [10,20,42]. Among these molecules, 3,6-nonadienol; 3-nonen-1-ol; and 1-nonanol were detected in the M_AI_V_t_0_ sample (Table 1), with a total amount of 6.40 × 10^7^ (peak TIC area), corresponding to ≈11.5% of the total area of the ALC group. They imparted a fresh, green-like aroma, and 3-nonen-1-ol is even called “melon aroma”.

It is believed that some of the C9 aromatic compounds of the *Cucurbitaceae* family may not be present in all melon cultivars due to genetic or varietal differences, post-harvest fruit handling, the degree of ripeness at harvest, the climate, and the cultivation method [32]. Furthermore, we can assume that some differences found in the flavor profile may be attributed to the extraction, sampling technique and chromatographic conditions applied. In any case, we observed a decrease in the overall quantity of alcohols passing from the crude fiber (M_AI_V_t_0_: 5.55 × 10^8^) to bleached one (M_B_O_t_0_: 1.33 × 10^8^) and, again, to the aged fiber (M_B_O_t_24_: 1.53 × 10^8^). This could be related to the development of analytes characterized by more oxidized functional groups, such as ketones and organic acids.

### 3.3. Ketones

As can be seen from Table 1, the M_AI_V_t_0_ fiber is characterized by the presence of 3-hydroxy-2-butanone (acetoin) and butanedione, detected in similar amounts based on the TIC area (6.45 × 10^7^ and 7.88 × 10^7^, respectively). Among the analytes belonging to this class of molecules, these two are found in the highest quantities in the previously mentioned sample. Acetoin has a pleasant creamy-yogurt smell, generally used in the food industry to enhance the flavor of some products. It has been identified in several dietary products such as yogurt and cheese, vinegar, fruits, vegetables, and some types of flours [43]. In relation to the category of melons, it has previously been detected in some cultivars in Italy [44]. It also has good biocompatibility and solubility, which extends its usage to soaps, lotions, and cosmetics [45]. In general, acetoin has three natural origins: (i) microbial fermentation, (ii) vegetable synthesis by fruits or plants, and (iii) animal synthesis. Its biosynthesis in plant cells was confirmed in the literature. It is also reported that, along with other odorous compounds, it confers to plants rich fragrances, with the purpose of attracting insects and higher animals to help with its pollination and propagation [43]. Being a very active molecule, acetoin generates many derivatives, which can be detected through HS-SPME-GC-MS analysis.

**Figure 6:**
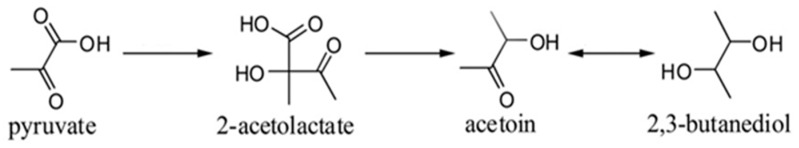
Scheme of acetoin and 2,3-butanediol biosynthetic pathway [34].

For example, 2,3-butanediol is a product of the acetoin pathway in many bacterial species. This diol is produced from pyruvate during fermentation processes via several intermediate metabolites, where different enzymes are involved. At present, the exact metabolic function of 2,3-butanediol is not well known. It may play a role in avoiding excessive intracellular acidification through the conversion of an acid compound (pyruvic acid) to a neutral one (acetoin) [45,46], as represented in Figure 6.

Butanedione is also produced at the beginning of fermentation processes by yeasts, but it is generally rapidly reduced to acetoin and 2,3-butanediol. Together with acetoin, the diol imparts a buttery aroma to the food in which it is contained [47]. In our case it is quite unlikely that fermentation processes took place markedly, given the speed of handling the melon pulp from the fresh fruit to the dried fiber. Thus, most of the acetoin could, instead, have had a natural origin. However, since all the metabolites and intermediates previously cited have been detected, it cannot be excluded, a priori, that some microorganism initiated the metabolic processes just described.

Among other ketones, 6,10-dimethylundeca-5,9-dien-2-one (geranylacetone), which was present only in the M_AI_V_t_0_ sample (Table 1), is known to be derived from the natural degradation of long-chain terpenes; these are typically β-carotene and lycopene, which give a floral aroma to the ripe fruit [10]. The same occurs for 6-methyl-5-hepten-2-one. In this case, given the pale yellowish-greenish color of the winter melon flesh, we suppose that it may originate from some other carotenoid or xanthophyll naturally present in this fruit and not from lycopene or β-carotene. These two molecules may also result from the degradation of ζ-carotene, a linear carotenoid with a weak yellow color [48,49]. Finally, among ketones, C8 compounds were the main ones in the HS of both the bleached fibers, with 2-octanone; 3-octen-2-one; and 3,5-octadien-2-one. The amounts of these analytes were about 35% and ≈43%, respectively, for the M_B_O_t_0_ and M_B_O_t_24_ samples (the relative area % only refers to the ketones class). In particular, 3,5-octadien-2-one was present in greatest quantities among the three above-mentioned species, existing in the form of two conformational isomers.

### 3.4. Esters

**Figure 7:**
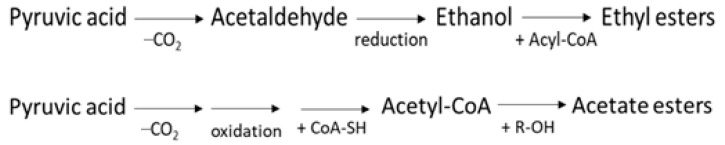
Scheme of the biosynthetic pathway which leads to the formation of ethyl and acetate esters, starting from pyruvic acid [25].

Fruit esters are generated by the action of enzymes and/or fermentative metabolism processes, which can be enhanced by various factors, such as maturation (intrinsic factors), microbial growth (biotic factors), as well as climate and seasonal temperature (extrinsic factors) [23]. The aroma quality assessment of 39 melon cultivars conducted by Shi et al. [10] showed that ethyl acetate was present in the highest amount among esters, followed by 2,3-butanediol diacetate. This perfectly agrees with our experimental results, where 2,3-butanediol diacetate was even detected as two stereoisomers. Additionally, Güler et al. identified ethyl acetate as the main ester in several varieties of melon, both climacteric and non-climacteric ones [32]. This volatile compound contributes to the complexity of the aroma and gives positive notes but, if present at too high a level, it becomes an off-flavor, conferring the typical “solvent” or “ethereal” odor to the over-ripened fruit. The biosynthetic pathway of a huge number of plant VOCs can be traced back to primary metabolism. For example, some aliphatic esters can be produced by the oxidation of free fatty acids—such as linoleic and linolenic acids—generating short-chain compounds, or starting from some amino acids such as valine and aminobutyric acid [28,32]. It is known that biochemical reactions are regulated by various factors such as physical conditions, kinetic factors, and enzymatic activity. As regards ester formation, it is generally believed that they can also be enzymatically formed by the combination of an alcohol with an acyl group. In particular, ethyl esters and acetate esters can be formed following two different biosynthetic pathways (Figure 7), but they have pyruvic acid as the same precursor [25].

Pyruvic acid is the key product of glycolysis, a biological process that occurs in the cytoplasm of the cell and that is present in all living organisms [50,51]. This process consists of a chain of reactions in which several enzymes are involved, and wherein glucose is converted to two molecules of pyruvic acid. In plants, glucose is constantly available in large quantities, as it is the final product of photosynthesis and represents the most suitable form of carbohydrate storage.

### 3.5. Acids

Organic acids mainly contributed to the chemical composition of the HS of the bleached fibrous sample, particularly the one aged two years. As can be seen from Table 3, in M_B_O_t_24_, we detected nine acids wherein the acetic, propanoic and hexanoic ones were present in higher quantities (peak TIC area) within this class of compounds. As for acetic acid, there was a progressive and significant increase in its amount, passing from the untreated fiber (M_AI_V_t_0_ : 5.34 × 10^7^) to the bleached ones, both for the sample at t_0_ time (M_B_O_t_0_ : 3.3 × 10^8^) and for the one aged 24 months (M_B_O_t_24_ : 8.2 × 10^8^). This trend was always associated with the total loss of 2,3-butanedione. Indeed, it has been shown that 2,3-butanedione reacts with H_2_O_2_ to give acetic anhydride, which is rapidly and completely hydrolyzed to acetic acid [33]. Since this is a reaction involving diketones and the total loss of 2,3-pentanedione also being detected, it is reasonable to hypothesize that this compound has also been completely oxidized to still give acetic and propanoic acids. In fact, a gradual and progressive increase in the quantity of the latter was observed as well. Finally, hexanoic acid is probably derived from the oxidation of some precursors, such as C6 alcohols and aldehydes.

### 3.6. Sulfur-Derived Compounds

Compounds containing sulfur can have a major impact on the overall flavor perception of fruits and vegetables. However, many of them are recognized as off-flavors of many foods due to their characteristic unpleasant odors, associated with a very low sensory detection threshold [35]. Off-notes typically increase in quantity as a consequence of heat treatment, such as sterilization techniques, used to ensure safety and extend the shelf-life of perishable foods [31,35]. The concept of off-flavor can be considered partly subjective, depending on various factors, such as the type of analyzed matrix, the quantity of analytes considered, and the impact that their characteristic aroma has on the entire aromatic component of the food. In addition, considering that only odor-active compounds contribute significantly to the unpleasant taste notes, in this case, it was only possible to compare this family of analytes with the literature data, without speculating on how pronounced their impact on the entire flavor profile of the investigated samples was.

In particular, we identified some sulfur compounds in the M_AI_V_t_0_ sample: dimethyl sulfide (DMS), dimethyl disulfide, dimethyl sulfoxide (DMSO), 3-methylthio-propanal, ethyl-methylthioacetate, 2,3-dihydro-thiophene and benzothiazole. Most of them have previously been identified in thermally treated melon juices [26,30,31,35]. It has also been reported that some of these compounds, particularly dimethyl sulfide and dimethyl disulfide, originate from the degradation of sulfur amino acids, such as methionine, cysteine, and their derivatives, when involved in some reactions induced by heat (i.e., Maillard reactions).

However, there is a big difference between our samples and those shown in the aforementioned papers. In our case, the fiber did not undergo any heat treatment, but was vacuum-freeze-dried, in order to preserve the original features of the pulp as much as possible. It was interesting to find that DMS, DMSO and, to a lesser extent, dimethyl sulfone are widely distributed in nature in various fruits; vegetables, including cucumber; forage; and also in some beverages [34]. In particular, DMS is responsible for the aroma of many foods and is supposed to play an important role in the natural transfer of sulfur of biological origin. Moreover, it has been shown in the literature that its photolysis in the presence of air generates DMSO [34]. However, it cannot be overlooked that DMSO is also widely used as an organic solvent to produce pesticides, and therefore, its presence could be partly due to some form of external contamination. In addition, we also detected 2,3-dihydrothiophene—which is known to contribute to the aroma of white truffle [52]—and benzothiazole. These sulfur compounds have been found in various foods such as persimmons, potatoes, and tea [52], but may also partially result from Maillard reactions [31]. Furthermore, ethyl-methylthio-acetate has also been previously found in various fruits, including melon [28,52]. Finally, we report that none of the sulfur compounds were detected in the H_2_O_2_-bleached fibrous samples.

### 3.7. Other Compounds

In this heterogeneous group, we collected the residual analytes, which did not seem to be related to the compounds identified and previously classified according to their molecular structure and functional groups. Even though some molecules such as methoxy-phenyl-oxime, pyrrole and pyranone, as well as epoxybutyrate, were detected in our samples and in other fruits, there was a lack of information on the chemical and biochemical cycles that rationalize their presence in nature. Finally, it is worth noting that for propylene glycol, identified in the M_AI_V_t_0_ sample (Table 1), we did not find mention of *Cucurbitaceae* fruits in the specific literature.

Table 4 summarizes the results obtained from the HS-SPME-GC-MS analysis for the different fibrous samples from winter melon pomace, collected in comparative form based on the classes of compounds identified.

A one-way analysis of variance (ANOVA) was performed to assess whether there was a statistically significant difference between the quantities of each compound class among the M_AI_V_t_0_, M_B_O_t_0_, and M_B_O_t_24_ samples. The *p*-value, for almost all the investigated compounds, excluded AHA and OTH groups, was smaller than the significance level (0.05). So, we can conclude that, globally, the investigated DF samples are statistically different.

For clarity, Figure 7 summarizes the results obtained from the HS-SPME-GC-MS analysis for the different fibrous samples of the winter melon pulp, grouped comparatively according to the classes of the identified compounds.

**Figure 8:**
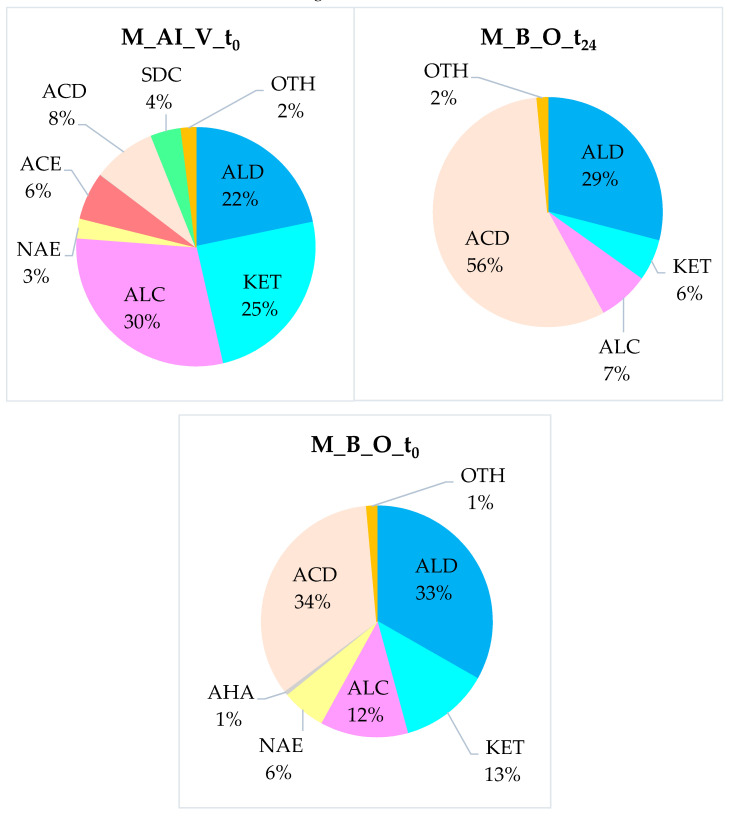
Graphical representation of the relative percentage area of the compound classes, identified in the HS-SPME-GC-MS analysis of the DF samples obtained from winter melon pomace.

The fibers obtained after the bleaching process with H_2_O_2_ at t_0_ time (M_ B_O_t_0_) and the sample stored for 24 months (M_B_O_t_24_) presented an HS composition significantly different from that of the “As-it-Is”, vacuum-freeze-dried pomace fiber (M_AI_V_t_0_). In particular, some specific considerations can be developed for some classes of molecules, listed in Table 4 and Figure 8.

(i) Volatile compounds typical of the ripe fruit (alcohols, esters): Alcohols covered about one third of the VOCs of the M_AI_V_t_0_ fiber, while immediately after the bleaching treatment, they represented ≈12 % of the volatiles (M_B_O_t_0_) and only ≈7 % in the aged sample (M_B_O_t_24_). Acetate esters were present only in the M_AI_V_t_0_ sample at a relative percentage of about 7 %, and completely disappeared in the treated ones. On the other hand, non-acetate esters persisted even after the chemical whitening process of the melon pomace fiber, reaching ≈6 % in the M_B_O_t_0_ sample, but not after the long storage period.
(ii) Volatiles completely lost during the bleaching process (Sulfur-Derived Compounds): We report that six SDCs were detected only in the crude fiber (M_AI_V_t_0_), at around 4.5%, while none of them were identified in the bleached ones. The total loss of this class of analytes may have been caused by their intrinsic volatility in combination with the hydrolysis processes in which they can be involved, due to being in the presence of organic acids and residual moisture. Finally, even the repeated washing of the fibers may have contributed to the removal of some analytes present in the “As-it-Is” pomace.
(iii) Molecules developed from the DF treatment with H_2_O_2_ (organic acids): The fraction of acids was the largest in the M_B_O_t_24_ sample (≈57%), while it covered only about 10% of the total volatile organic fraction of the M_AI_V_t_0_ and reached ≈34% in the M_B_O_t_0_ ones. Certainly, the action of H_2_O_2_ strongly contributed to the development of these analytes, oxidizing some of the molecules originally present in the starting matrix, to give products with acid functional groups. In addition, the oxygen present in the residual atmosphere of the preservation vials also led to an increasing quantity of organic acids over time, with O_2_ acting as a starter for radical oxidation processes.

In general, it is quite evident that the bleaching of the fiber from winter melon pomace, and the aging even more so, leads to a simplification of its HS aromatic bouquet. In fact, there is a progressive reduction in the classes of molecules detected, passing from the initial nine groups to four after only 24 months.

## 4. Materials and Methods

### 4.1. Sample Preparation

Melons, produced in Sicily (Italy), were purchased at a local supermarket in Modena city. Five different fruits were taken and considered, in order to give a representative analytical sample. The melons were ready to use and in ripe condition at the time of purchase.

**Figure 9:**
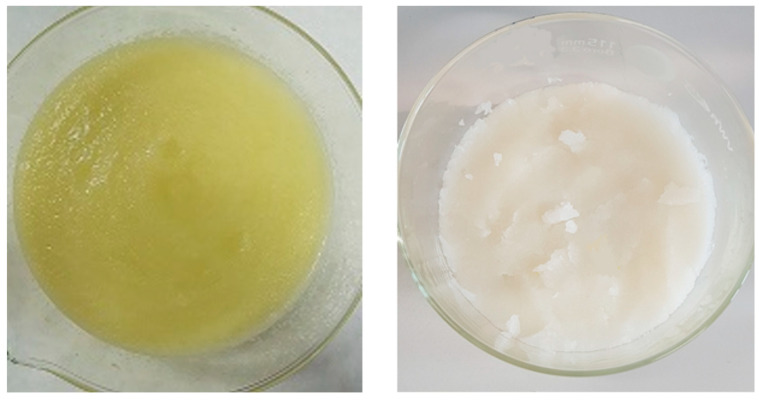
Pulp slurry-meal from winter-melon (*Cucumis Inodorous*): natural (**left**) and bleached pomace (**right**).

The melons were washed with distilled water, peeled, and deseeded. Three slices of about 100 g each were taken from different orientations of each fruit and used for one composite sample. The white pulp was shredded using a kitchen mixer to obtain a homogeneous slurry-meal (Figure 9).

The pulp was then filtered with paper to separate most of the juice from the fibrous fraction, which was finally divided in two aliquots: (i) one of them was vacuum-freeze dried (CHRIEST, Mod. Alpha 1-2 LDplus; Direct Industry, Osterode am Harz, Germany) for 20 h, 15 of which were at 1 mbar, and the remaining at 0.001 mbar, to obtain the raw fiber of the whole meal (sample M_AI_V); (ii) the other fraction was bleached with food-grade H_2_O_2_ and then oven dried at 40 °C for 48 h (sample M_B_O).

More specifically, the latter was obtained by keeping the polysaccharide material under constant stirring for 2 h, at room temperature, after the addition of H_2_O_2_ (conc. 10%) at a ratio of 3:1 (fiber: H_2_O_2_) m/m. Finally, it was rapidly filtered again, and the fibrous fraction was oven dried as previously specified.

Immediately after the drying process for both samples, the resulting fibers were homogenized in a grinding mill equipped with a rotor made in Ti and a sieve of 500 μm; the equipment was maintained at a low temperature by refrigerating it with a few drops of liquid N_2_. This strategy was adopted in order to preserve, as much as possible, the aromatic characteristics of the fibers. Then, a consistent set of samples was prepared as follows: about 0.5 g of material was transferred to 10 mL glass vials, which were sealed tightly with Teflon/silicone septa.

The vacuum-freeze-dried specimens were immediately analyzed after they were prepared (at t_0_ time), in order to characterize the VOCs’ aromatic fraction of the starting material (M_AI_V_t_0_). Contrarily, the bleached fiber was analyzed at the t_0_ time (M_B_O_t_0_) and after a storage period of 24 months (M_B_O_t_24_), preserving the samples in the dark, at room temperature, and in the same closed vials, without ever opening them during the aging period.

All the analyses were carried out at least in triplicate, as described in the following sections.

### 4.2. Volatile Organic Compounds Sampling—HS SPME

A Solid-Phase Micro-Extraction (SPME) holder (Supelco Inc., Bellefonte, PA, USA) was used to manually perform the SPME headspace (HS) analysis.

Before being analyzed, all the samples were sonicated for 30 min in a thermostated bath at 40.0 ± 0.1 °C to favor the transfer of volatile compounds from the matrix to the headspace. After this step, the extraction of volatiles was performed by manually exposing a 2 cm-long SPME fiber composed of CW/DVB/PDMSO (Supelco Inc, Bellefonte, PA, USA) to the HS of the vial for 15 min, at the same temperature. Finally, the fiber was withdrawn and inserted into the injector port of a GC-MS system for desorption of the analytes at 250 °C for 15 min. Given the characteristics of different polarity of the 3 constituents of the fiber, this device seemed suitable for the capture of analytes with significantly different molecular properties and dimensions, making it adequate for the characterization of complex matrices, as in this case.

The reproducibility of experimental procedures was established by working with at least three replicate samples of the same matrix and taking many different measures for each vial.

Some blank tests, corresponding to the analysis of an external standard solution containing 1-decanol (conc. 150 µg/g ethanolic solution), were performed periodically after a certain number of chromatographic runs (five) relating to real samples.

### 4.3. GC-MS Analysis

GC-MS analysis was performed using an Agilent 6890N Network gas chromatograph system coupled with a 5973N mass spectrometer (Agilent Technologies, Santa Clara, CA, USA). A DB-5MS UI column (60 m × 0.25 mm i.d., 1.00 μm film thickness; J&W Scientific, Folsom, CA, USA) was used for chromatographic separation. The SPME injections were performed in splitless mode, and the detector started to operate immediately after each injection. The carrier gas (He) was fluxed at a constant flow of 1 mL/min, with a column head pressure of 15 psi. The initial oven temperature was 40 °C (held for 5 min), followed by a heating ramp set at 10 °C/min up to 160 °C, and then 8 °C/min, to reach the final temperature of 270 °C, held for 5 min. The transfer line was heated to 270 °C.

The mass spectrometer was operated in electron impact (EI) ionization mode at 70 eV, in full-scan acquisition mode, with a *m/z* scanning range from 25 to 300.

Chromatograms and mass spectra were analyzed using the Enhanced ChemStation software (Agilent Technologies, Santa Clara, CA, USA). Tentative identification of volatile compounds was achieved by matching the mass spectra with the data system library (NIST14 / NIST05 / WILEY275 / NBS75K) and by using some databases accessible via the web, such as the National Institute for Standards and Technology (NIST database https://webbook.nist.gov, accessed on 1 September 2021) and the Mass Bank of North America (https://mona.fiehnlab.ucdavis.edu, accessed on 1 September 2021).

A Linear Retention Index (LRI) was used for an additional comparison between our data and those reported in the literature and in the NIST Standard Reference Database, only considering values referring to analyses carried out under the same operative conditions (instrumental specifications and heating ramp).

The LRIs of compounds were calculated from a series of n-alkanes (C6, C9, C12, C14, C16) subjected to the same analysis procedure as that adopted for the samples.

The latter proved to be particularly useful for the distinction of E/Z isomers, since these species produce mass spectra that are difficult to differentiate.

Finally, some analytes were identified by comparing their mass spectra with those of their respective pure standards (when available), analyzed using HS-SPME-GC-MS under the same operating conditions as those used for the samples.

The volatile compounds such as silane and siloxane derivatives, or Volatile Organic Compounds related to the sorbent fiber, were discarded and are not reported in the GC-MS output tables. The estimation of the amount of each volatile identified in the SPME-GC-MS analysis was expressed as the Total Ion Current (TIC) peak area.

All the data shown in the tables relate to values obtained from analysis performed at least in triplicate. The reproducibility of the results is expressed as standard deviation in the tables. When absent, the apex ^a^ indicates that SD < 0.05.

### 4.4. Chemicals and Reagents

The chemicals used during the study were: (i) 2-methyl-1-propanol, 1-pentanol; 2-ethyl-1-hexanol, phenylethyl alcohol, 2-phenoxyethanol, 3-methylbutanal, 2-ethyl-2-butenal, ethyl butanoate, ethyl-3-hydroxybutanoate, 3-hydroxy-2-butanone (acetoin), 2-methylbutanoic acid, and benzothiazole, and were obtained from Sigma-Aldrich, distributed by Merck KGaA, Darmstadt, Germany; and (ii) decanal, methyl acetate, 1-decanol, n-hexane, nonane, dodecane, tetradecane and hexadecane, which were obtained from Carlo Erba Reagents, Milano (Italy).

### 4.5. Statistical Analysis

The experimental data were compared by conducting an analysis of variance (one-way ANOVA), using the Matlab^®^ 2019b environment (Mathworks Inc., Natick, MA, USA). The level of significance was determined at *p* < 0.05, to see whether there were statistical differences between the mean values.

## 5. Conclusions

The results of this paper clearly show the validity of the proposed analytical method of VOC sampling and chemical characterization of the DF obtained from the *Inodorous* melon. HS-SPME is an extremely high-performance technique and provides highly reproducible results, even for analytes present in low amounts. Indeed, this method is suitable for the effective screening of a wide variety of volatile and semi-volatile compounds related to the aromatic properties of the analyzed materials.

Different groups of analytes allowed for discrimination of the investigated fiber samples, according to preliminary treatments, as well as aging of the DF. Furthermore, it was possible to highlight that the bleaching treatment with H_2_O_2_ reduced the number of VOC groups from 9 to 7, detected at the t_0_ time of sample preparation. Conversely, an increment of the quantitative ratios of the oxidized molecules, such as acids—at the detriment of alcohols and ketones—was observed in the bleaching treatment samples. These composition ratios progressively changed and increased even during the fiber aging, reducing the compound classes to four at t_24_ months of shelf-life.

The obtained results could be particularly useful in identifying some target compounds as qualitative and quantitative markers, and can increase knowledge on the influence of treatments and aging time on final compositions of samples from differently treated *Inodorus* melon fibers.

For example, acetic esters (ACE), pleasant aromatic molecules, were always present in ‘As-it-Is’ samples, but they did not persist upon treatment with H_2_O_2_. On the contrary, non-acetic ester (NAE) compounds survived to bleaching treatment but disappeared after the long aging time. As far as the aldehyde group (ALD) is concerned, it was possible to note its stability and durability over time, especially for the representative compounds of the C6 subclass. Finally, organic sulfur compounds (SDCs) were identified among the groups of molecules that were not resistant to bleaching treatment; indeed, they are generally classified as off-flavor. Therefore, their elimination could have a very positive effect on the final characteristics of the fiber.

These results surely represent a benchmark for the application of the proposed method to the study of other vegetable matrices from the *Cucurbitaceae* family. The possibility of obtaining DF-enriched products represents an important opportunity to meet the growing market demand for dietary fiber and, at the same time, to enhance the recovery of agro-industrial pre-waste materials. Finally, the potential inclusion of this ingredient in many foods may change the needs of processing industries and consumers, as well as enabling more widespread food sustainability.

## Figures and Tables

**Table 1:** VOC composition of vacuum-freeze-dried melon pomace, at t_0_ time (M_AI_V_t_0_) identified through HS-SPME-GC-MS analysis, grouped by chemical classes. Data are expressed as mean (*n* = 3) TIC area × 10^7^ ± SD_(3)_.

Compound	LRI	ID ^#^	Aroma	Area	Ref
***Alcohols***					
1-Propanol	538	A, B	Alcoholic, fermented	0.22 ^a^	-
2-methyl-1-Propanol	639	A, B, C	Ethereal	0.24 ^a^	[19]
1-Penten-3-ol	720	A, B	Ethereal, green, vegetable	0.74 ^a^	[20,21]
Propylene glycol	789	A, B	-	1.2 ± 0.1	[22,23]
1-Pentanol	829	A, B, C	Pungent	0.78 ^a^	[20,21,24,25,26]
(*R*,*R*)-2,3-Butanediol	845	A, B, D	Creamy, fruity, buttery	7.4 ± 0.2	[26,27]
(*R*,*S*)-2,3-Butanediol	858	A, B, D	Creamy, fruity, buttery	20.0 ± 0.3	[26,27]
3-Pentanol	875	A, B	Sweet, herbal, oily, nutty	5.19 ± 0.1	[20,21,24,25,26]
3-Hexen-1-ol	937	A, B	Green, leafy	1.2 ± 0.1	[10,28,29]
1-Hexanol	948	A, B	Green, flowery	0.53 ^a^	[10,20,24,26,28,30]
1-acetoxy-2-Propanol	955	A, B	-	0.67 ^a^	-
2-acetoxy-1-Propanol	967	A, B	-	0.49 ^a^	-
1-Octen-3-ol	1065	A, B	Mushroom, earthy, green	2.4 ± 0.1	[10,24,29]
2-ethyl-1-Hexanol	1112	A, B, C	Citrus, fresh, floral, sweet	0.63 ^a^	[10,20]
Benzyl alcohol	1131	A, B	Sweet, floral, fruity	4.9 ± 0.2	[10,22,24,29,31]
Phenylethyl alcohol	1212	A, B, C	Floral, sweet	1.4 ± 0.1	[10,31]
2,6-dimethyl-Cyclohexanol	1216	A, B	-	0.72 ^a^	[19]
3-Nonen-1-ol	1233	A, B	Green, melon	1.9 ± 0.1	[10,20,21,29,31]
3,6-Nonadienol	1236	A, B	Fresh, green, melon, cucumber	2.7 ± 0.1	[10,24,28]
1-Nonanol	1243	A, B	Fresh, fatty, floral	1.8 ± 0.1	[10,21,24,29,31]
2-Phenoxyethanol	1306	A, B, C	Floral, balsamic	0.46 ^a^	[31]
***Aldehydes***					
Propanal	458	A, B	Ethereal, pungent, earthy, alcoholic	0.64 ^a^	[24]
2-methyl-Propanal	543	A, B	Malty	0.49 ^a^	[19,31]
2-Butenal	675	A, B	Flower	19.8 ± 0.3	[20]
3-methyl-Butanal	685	A, B, C	Fruity, green, cocoa	1.3 ± 0.1	[20,29,30,31]
2-methyl-Butanal	698	A, B	Malty, cocoa, almond	0.90 ^a^	[19,20,30,31]
Pentanal (valeraldehyde)	742	A, B	Fermented, bready, almond, malt	2.1 ± 0.1	[24,26,29,30,32]
2-methyl-2-Butenal	805	A, B	Pungent, green, ethereal	2.9 ± 0.1	-
2-ethyl-2-Butenal	899	A, B, C	-	0.35 ^a^	-
Furfural	918	A, B	Woody, almond, sweet, fruity, floral	0.63 ^a^	[19]
2-ethenyl-2-Butenal	930	A, B	-	1.01 ^a^	-
Heptanal	988	A, B	Fresh, fatty, green, herbal	0.55 ^a^	[10,20,21,26,29,30]
2-ethyl-3-methyl-Butanal	992	A, B	-	0.45 ^a^	
2,4-Hexadienal	1002	A, B	Fatty, sweet, green	0.39 ^a^	[29]
2-Heptenal	1047	A, B	Green, fatty	1.6 ± 0.1	[10,20,21,24,29]
Benzaldehyde	1069	A, B	Bitter, almond-like, fruity	0.7 ± 0.1	[10,20,22,24,28,30]
Benzeneacetaldehyde	1146	A, B	Green, floral, honey	1.0 ± 0.1	[10,24,31]
Nonanal	1187	A, B	Waxy, fresh, green, citrus	3.2 ± 0.1	[10,20,24,29,30]
Decanal	1279	A, B, C	Sweet, waxy, citrus, green melon	1.0 ± 0.1	[10,20,24,29]
Benzeneacetaldehyde, α-ethylidene	1353	A, B	-	1.6 ± 0.1	-
***Esters***					
Methyl acetate	499	A, B, C	Ethereal, fruity	0.32 ^a^	[10,24,25,32]
Ethyl acetate	616	A, B	Ethereal, fruity, sweet	7.2 ± 0.2	[10,24,25,29,32]
Ethyl butanoate	869	A, B, C	Fruity	0.8 ± 0.1	[10,22,24,31]
Ethyl 2-methyl-Butanoate	926	A, B	Fruity	1.0 ± 0.1	[31]
Ethyl 3-hydroxy-Butanoate	1019	A, B, C	-	1.2 ± 0.1	-
1-Butanol,3-methyl-, propanoate	1061	A, B	-	0.62 ^a^	-
Ethyl 2,3-epoxybutyrate	1098	A, B	-	1.4 ± 0.1	[23]
2,3-Butanediol diacetate	1121	A, B	Earthy, soil-like odor	2.2 ± 0.1	[24,28]
2,3-Butanediol diacetate	1134	A, B	Earthy, soil-like odor	2.2 ± 0.1	[24,28]
***Ketones***					
Acetone	454	A, B	Solvent, ethereal	0.46 ^a^	[30]
2,3-Butanedione	582	A, B	Buttery, sweet, creamy	7.9 ± 0.3	[31,33]
2-Pentanone	590	A, B	Sweet, fruity, ethereal	0.58 ^a^	-
1-hydroxy-2-Propanone	691	A, B	Sweet, caramel-like	0.8 ± 0.1	-
2,3-Pentanedione	735	A, B	Buttery, sweet, caramel-like	2.1 ± 0.1	[19,31,33]
3-hydroxy-2-Butanone (Acetoin)	755	A, B, C	Acid, yogurt, creamy, fruity	6.4 ± 0.2	[22,29]
3-Penten-2-one	795	A, B	Fruity	3.2 ± 0.2	-
4-hydroxy-5-methyl-2-Hexanone	964	A, B	-	0.30 ^a^	-
***Ketones (Continued)***					
6-methyl-5-Hepten-2-one	1070	A, B	Citrus, green, fruity	1.0 ± 0.1	[10,20,29]
3,5-Octadien-2-one	1155	A, B	Fruity, fatty, mushroom	0.62 ^a^	[24,29,31]
Geranylacetone	1484	A, B	Floral, fresh, green	0.27 ^a^	[10,31]
***Acids***					
Acetic acid	571	A, B	Sour, pungent	5.3 ± 0.2	[22,29,31]
Propanoic acid	709	A, B	Pungent, acidic	1.1 ^a^	[33]
2-methyl-Propanoic acid	793	A, B	Acidic, sour	0.50 ^a^	[19]
3-methyl-Butanoic acid	897	A, B	Sour, cheesy	1.3 ± 0.1	-
2-methyl-Butanoic acid	909	A, B, C	Cheesy	2.1 ± 0.1	[22,31]
2-Hydroxy-2-methylbutyric acid	958	A, B	-	3.5 ± 0.2	-
Hexanoic acid	1040	A, B	Sour, fatty, sweet, cheesy	1.8 ± 0.1	[33]
Nonanoic acid	1312	A, B	Waxy, cheesy, dairy	0.34 ^a^	[10]
***Sulfur-derived compounds***					
Dimethyl sulfide	492	A, B	Sulfurous, onion, corn, vegetable	4.7 ± 0.2	[30,31,34,35]
Dimethyl disulfide	816	A, B	Sulfurous, onion, cabbage	0.73 ^a^	[25,26,30,31,35]
Dimethyl Sulfoxide	923	A, B	Fatty, oily, cheesy	0.26 ^a^	[34]
Methional	998	A, B	Cooked potato, earthy, vegetable	0.58 ^a^	[10,22,29,31]
Ethyl (methylthio)acetate	1073	A, B	Fruity, sulfurous, green	0.24 ^a^	[10,24]
2,3-dihydro-Thiophene	1298	A, B	-	1.2 ± 0.1	-
***Alkanes***					
Tridecane	1269	A, B	-	0.16 ^a^	[31]
Tetradecane	1438	A, B, C	Waxy	0.11 ^a^	[24,28]
***Others***					
Methoxy-phenyl-oxime	950	A, B	-	0.6 ± 0.1	-
3-ethyl-2,5-dimethyl-Pyrazine	1167	A, B	Nutty, potato, cocoa, rosted	0.98 ^a^	-
2,3-dihydro-3,5-dihydroxy-6-methyl-4H-Pyran-4-one	1240	A, B	-	1.6 ± 0.1	[26,36,37,38]
Benzothiazole	1338	A, B, C	vegetable, cooked, coffe-like	0.24 ^a^	-
1-Tridecene	1499	A, B		0.20 ^a^	[38]

^#^ The identification is indicated by: (A) mass spectral data of the libraries supplied with the operating system of the GC-MS and from mass spectra databases; (B) mass spectra found in the literature; (C) mass spectra and retention time of an injected standard; (D) LRI values, typically used for the identification of isomers. ^a^ SD < 0.05.

**Table 2:** VOC composition of bleached melon pomace at t_0_ time (M_B_O_t_0_), identified through HS-SPME-GC-MS analysis, grouped by chemical classes. Data are expressed as mean (*n* = 3) TIC area × 10^6^ ± SD_(3)_.

Compound	LRI	ID ^#^	Aroma	Area	REF
***Alcohols***					
1-Penten-3-ol	720	A, B	Ethereal, green, vegetable	14.9 ± 0.2	[20,21,22]
1-Pentanol	829	A, B, C	Pungent,	7.6 ± 0.1	[10,20,24,25,26]
(*R*,*R*)-2,3-Butanediol	845	A, B, D	Creamy, fruity, buttery	19.4 ± 0.3	[26,27]
(*R*,*S*)-2,3-Butanediol	857	A, B, D	Creamy, fruity, buttery	71.4 ± 0.4	[26,27]
2-Furanmethanol	933	A, B	Alcoholic, bready, caramel-like	1.7 ^a^	-
1-Octen-3-ol	1065	A, B	Mushroom, earthy, green	10.4 ± 0.2	[10,24,29]
2-ethyl-Hexanol	1112	A, B, C	Citrus, fresh, floral, sweet	4.2 ^a^	[10,20]
2,6-dimethyl-Cyclohexanol	1215	A, B	-	3.69 ^a^	[19]
***Aldehydes***					
Propanal	458	A, B	Ethereal, pungent, alcoholic	9.6 ± 0.2	[24]
2-methyl-Propanal	543	A, B	Malty	2.11 ^a^	[19,20]
2-Butenal	677	A, B	Flower	2.03 ^a^	[20]
3-methyl-Butanal	685	A, B, C	Fruity, green, cocoa	10.2 ± 0.3	[20,29,30,31]
2-methyl-Butanal	698	A, B	Malty, cocoa, almond	3.66 ^a^	[19,20,31]
Pentanal (valeraldehyde)	742	A, B	Fermented, bread, almond, malt	58.6 ± 0.4	[19,24,26,29,32]
Hexanal	874	A, B	Grass, green, fat	155 ± 1	[10,19,20,21,22,23,24,25,26,27,28,29,30,31,32]
Furfural	916	A, B	Wood, almond, sweet, fruit	26.6 ± 0.4	[19]
Heptanal	988	A, B	Fresh, fatty, green, herbal	24.5 ± 0.5	[10,20,21,24,26,29,30]
2-Heptenl	1047	A, B	Green, fatty	5.1 ± 0.2	[10,20,21,24,29,31]
5-methyl-2-Furancarboxaldehyde	1056	A, B	Sweet, caramel-like, spicy	3.9 ± 0.1	-
Octanal	1091	A, B	Waxy, fatty, citrus, green	11.1 ± 0.3	[10,24,26,31]
2,4-Heptadienal	1104	A, B	Green, pungent, fruity, spicy	1.89 ^a^	-
Benzeneacetaldehyde	1146	A, B	Green, floral, honey	3.4 ± 0.1	[10,24,31]
Nonanal	1187	A, B	Waxy, fresh, green, cucumber	30.5 ± 0.5	[10,20,24,29,30]
Decanal	1279	A, B, C	Sweet, waxy, green, melon	12.4 ± 0.3	[10,20,24,29]
***Esters***					
2-oxo-Propanoic acid, methyl ester	776	A, B	-	59.9 ± 0.4	-
Isopropyl 3-methylbutanoate	1134	A, B	-	6.5 ± 0.2	[24,28]
***Ketones***					
Acetone	453	A, B	Solvent, ethereal	30.3 ± 0.4	[30]
1-hydroxy-2-Propanone	690	A, B	Sweet, caramel-like	10.6 ± 0.2	-
3-hydroxy-2-Butanone (Acetoin)	756	A, B, C	Acid, yogurt, creamy, fruity	5.7 ± 0.1	[22,29]
3-Penten-2-one	796	A, B	Fruity	4.2 ± 0.1	-
1-(acetyloxy)-2-Propanone	939	A, B	Fruity, buttery	8.7 ± 0.2	[28]
2-Heptanone	973	A, B	Fruity, spicy, sweet, herbal	12.8 ± 0.3	[29]
6-methyl-5-Hepten-2-one	1070	A, B	Citrus, green, fruity	14.8 ± 0.4	[10,20,29]
2-Octanone	1075	A, B	Earthy, woody, herbal	3.20 ^a^	-
3-Octen-2-one	1124	A, B	Earthy, herbal, spicy	13.6 ± 0.4	-
(*E*,*E*)-3,5-Octadien-2-one	1155	A, B, D	Fruity, fatty, mushroom	20.2 ± 0.5	[24,29,31]
(*E*,*Z*)-3,5-Octadien-2-one	1180	A, B, D	Fruity, fatty, mushroom	11.2 ± 0.2	[24,29,31]
***Acids***					
Acetic acid	591	A, B	Sour, pungent	326 ± 2.4	[22,29,31,33]
Propanoic acid	713	A, B	Pungent, acidic	22.3 ± 0.5	-
2-methyl-Propanoic acid	793	A, B	Acidic, sour	1.69 ^a^	-
3-methyl-Butanoic acid	896	A, B	Sour, cheesy	4.8 ± 0.1	-
Hexanoic acid	1041	A, B	Sour, fatty, sweet, cheesy	12.3 ± 0.3	-
2-methyl-Propanoic acid	793	A, B	Acidic, sour	1.69 ^a^	-
3-methyl-Butanoic acid	896	A, B	Sour, cheesy	4.8 ± 0.1	-
Hexanoic acid	1041	A, B	Sour, fatty, sweet, cheesy	12.3 ± 0.3	-
***Alkanes***					
Tridecane	1269	A, B	-	3.7 ± 0.1	[19]
3-methyl-Dodecane	1355	A, B	-	0.73 ^a^	-
Tetradecane	1438	A, B, C	Waxy	1.96 ^a^	[24,28]
***Others***					
Pyrrole	812	A, B	Sweet, nutty	3.6 ± 0.1	-
Methoxy-phenyl-oxime	951	A, B	-	4.6 ± 0.2	-
2(5H)-Furanone	1001	A, B	Buttery	3.0 ± 0.1	[31]
2-pentyl-Furan	1081	A, B	Fruity, green, earthy	3.1 ± 0.1	[20,24,26,29,30]
1-Tridecene	1499	A, B	-	1.12 ^a^	[28]

^#^ The identification is indicated by: (A) mass spectral data of the libraries supplied with the operating system of the GC-Ms and from mass spectra databases; (B) mass spectra found in the literature; (C) mass spectra and retention time of an injected standard; (D) LRI values, typically used for the identification of isomers. ^a^ SD < 0.05.

**Table 3:** VOCs of the bleached melon pomace after 24 months of storage (M_B_O_t_24_) identified through HS-SPME-GC-MS analysis, grouped by chemical classes. Data are expressed as mean (*n* = 3) TIC area × 10^6^ ± SD_(3)_.

Compound	LRI	Identification ^#^	Aroma	Area	REF
***Alcohols***					
1-Pentanol	831	A, B, C	Pungent	34.1 ± 0.4	[20,21,24,25,26]
(*R*,*R*)-2,3-Butanediol	848	A, B, D	Creamy, fruity, buttery, green, leafy	16.6 ± 0.3	[26,27]
(*R*,*S*)-2,3-Butanediol	859	A, B, D	Creamy, fruity, buttery, green, leafy	45.8 ± 0.5	[26,27]
1-Octen-3-ol	1065	A, B	Mushroom, earthy, green	49.5 ± 0.6	[10,24,29]
2-ethyl-1-Hexanol	1112	A, B, C	Citrus, fresh, floral, sweet	5.3 ± 0.1	[10,20]
2,6-dimethyl-Cyclohexanol	1215	A, B		1.62 ^a^	[19]
***Aldehydes***					
3-methyl-Butanal	688	A, B, C	Fruity, cocoa, green	28.5 ± 0.4	[20,29,30,31]
2-methyl-Butanal	701	A, B	Malty, cocoa, almond	9.4 ± 0.2	[19,20,30,31]
Pentanal	744	A, B	Fermented, bready, almond, malt	64.0 ± 0.5	[24,26,29,30,32]
Hexanal	876	A, B	Grass, green, fat	448 ± 4.8	[10,24,26,30,31,32]
Furfural	918	A, B	Woody, almond, sweet, fruity, floral	9.2 ± 0.3	[19]
2-Hexenal	938	A, B	Green, fruity, pungent, vegetable-like	13.1 ± 0.3	-
Heptanal	988	A, B	Fresh, fatty, green, herbal	35.4 ± 0.4	[10,20,21,24,29,30]
Octanal	1091	A, B	Waxy, fatty, citrus, green	13.1 ± 0.3	[10,24,26,31]
***Ketones***					
Acetone	464	A, B	Solvent, ethereal	32.9 ± 0.4	[30]
2-Heptanone	973	A, B	Fruity, spicy, sweet, herbal	30.2 ± 0.5	[29]
2-Octanone	1075	A, B	Earthy, woody, herbal	6.8 ± 0.1	-
3-Octen-2-one	1124	A, B	Earthy, herbal, spicy	21.2 ± 0.4	-
(*E*,*E*)-3,5-Octadien-2-one	1155	A, B, D	Fruity, fatty, mushroom	13.3 ± 0.3	[24,29,31]
Acetophenone	1170	A, B	Sweet, pungent, almond	8.4 ± 0.2	
(*E*,*Z*)-3,5-Octadien-2-one	1179	A, B, D	Fruity, fatty, mushroom	12.2 ± 0.28	[24,29,31]
***Acids***					
Formic acid	477	A, B	Pungent, vinegar	21.3 ± 0.4	
Acetic acid	630	A, B	Sour, pungent	823 ± 6.5	[22,29,31]
Propanoic acid	736	A, B	Pungent, acidic	143 ± 2.5	[33]
2-methyl-Propanoic acid	802	A, B	Acidic, sour	12.2 ± 0.3	-
3-methyl-Butanoic acid	900	A, B	Sour, cheesy	13.9 ± 0.3	-
2-methyl-Butanoic acid	911	A, B, C	Cheesy	10.4 ± 0.2	[22,31]
Pentanoic acid	945	A, B	Acidic, cheesy	29.5 ± 0.5	-
Hexanoic acid	1048	A, B	Sour, fatty, sweet, cheesy	137 ± 1.8	[22]
Heptanoic acid	1134	A, B	Sour, cheesy	16.2 ± 0.4	-

^#^ The identification is indicated by: (A) mass spectral data of the libraries supplied with the operating system of the GC-Ms and from mass spectra databases; (B) mass spectra found in the literature; (C) mass spectra and retention time of an injected standard; (D) LRI values, typically used for the identification of isomers. ^a^ SD < 0.05.

**Table 4:** Compound classes identified in the HS-SPME-GC-MS analysis * of the fiber samples obtained from winter melon pomace.

Compound Class	M_AI_V_t_0_	M_B_O_t_0_	M_B_O_t_24_	*p*-Value
	${\bar{x}}^{\;*}\;\pm \;s_{\left(3\right)}$	${\bar{x}}^{\;*}\;\pm \;s_{\left(3\right)}$	${\bar{x}}^{\;*}\;\pm \;s_{\left(3\right)}$	
ALC (alcohols)	33.8 ± 3.2	12.3 ± 2.3	7.3 ± 0.9	*p* < 0.05
ALD (aldehydes)	24.7 ± 2.4	33.2 ± 3.9	29.5 ± 2.7	*p* < 0.05
KET (ketones)	14.5 ± 2.6	12.5 ± 2.2	5.9 ± 0.8	*p* < 0.05
ACD (acids)	9.7 ± 1.7	33.8 ± 3.7	57.3 ± 4.4	*p* < 0.05
ACE (acetate esters)	7.2 ± 1.0	---	---	
SDC (sulfur compounds)	4.7 ± 0.8	---	---	
NAE (non-acetate esters)	3.0 ± 0.6	6.1 ± 0.7	---	*p* < 0.05
AHA (alkanes)	0.2 ± 0.2	0.6 ± 0.4	---	*p* > 0.05
OTH (other compounds)	2.2 ± 0.5	1.4 ± 0.9	---	*p* > 0.05

Data are expressed as mean % of each class to the total normalized peak areas on the basis of the sum of the TIC area. * Mean of three replicates of the chromatograms ± standard deviation *s*_(3)._

## Data Availability

Data is contained within the article.

## Sample Availability

Samples of the compounds are available from the authors.
